# Internet Use and eHealth Literacy of Low-Income Parents Whose Children Have Special Health Care Needs

**DOI:** 10.2196/jmir.1697

**Published:** 2011-09-29

**Authors:** Caprice Knapp, Vanessa Madden, Hua Wang, Phyllis Sloyer, Elizabeth Shenkman

**Affiliations:** ^1^University of FloridaDepartment of Health Outcomes and PolicyGainesville, FLUnited States; ^2^Florida Department of HealthChildren's Medical Services NetworkTallahassee, FLUnited States

**Keywords:** Children, Internet, Medicaid

## Abstract

**Background:**

The Internet has revolutionized the way in which many Americans search for health care information. Unfortunately, being able to use the Internet for this purpose is predicated on having access to the Internet and being able to understand and comprehend online health information. This is especially important for parents of children with special health care needs who are forced to make many medical decisions throughout the lives of their children. Yet, no information is available about this vulnerable group.

**Objective:**

For parents of children with special health care needs we sought to (1) describe their Internet access and use, (2) determine which child and household factors were associated with Internet use, (3) describe eHealth literacy of Internet users, and (4) determine which child and household factors were associated with greater eHealth literacy.

**Methods:**

This was a cross-sectional telephone survey of 2371 parents whose children with special health care needs were enrolled in Florida’s Medicaid and State Children’s Health Insurance Plan (SCHIP) programs (4072 parents were approached). To be enrolled in the program, families must have incomes that are less than or equal to 200% of the federal poverty level. The eHealth Literacy Scale (eHEALS) was used to measure eHealth literacy. Descriptive and multivariate analyses were conducted to address the study objectives.

**Results:**

The survey response rate was 58.2%. Participating parents were mainly female (2154/2371, 91%), white non-Hispanic (915/2371, 39%), English speaking (1827/2371, 77%), high school graduates (721/2371, 30%), married (1252/2371, 53%), and living in a two-parent household (1212/2371, 51%). Additionally, 82% of parents (1945/2371) in the sample reported that they used the Internet, and 49% of those parents used it daily (1158/2371). Almost three-quarters of Internet users had access to the Internet at home while about one-half had access at work. Parents who were African American, non-English speaking, older, and not college graduates were less likely to use the Internet than their referent groups (*P* < .001). About 74% of Internet users (1448/1945) reported that they knew how to find health information for their children. However, only about one-half (1030/1945) reported that they can tell high quality from low quality resources online or that they feel confident in using information accessed online to make health decisions. Multivariate regression results consistently showed that being a non-English speaker, having less than a high school education, and being older were all significantly associated with lower eHealth literacy (all *P* < .001).

**Conclusion:**

Low-income parents of children with special health care needs have access to and use the Internet as a source of information about their children's health. However, some parents are unable to distinguish between high and low quality information and are not confident in using the Internet. This information is timely because as the pressure to use the Internet to empower consumers and exchange information increases, issues related to access and disparities must be better understood.

## Introduction

There is no doubt that the World Wide Web has significantly impacted the world since the mid-1990s. Information that was once available to only those with time, money, and knowledge is now available at the click of a button to those who have access to the Internet. The Pew Research Center’s Pew Internet and American Life Project is perhaps the most comprehensive series of research on how access to, and use of, the Internet has evolved since the early 2000s. The Pew Research Center’s first report in 2000 focused on how women used the Internet. Findings from the report noted that about 26 million Americans had used the Internet to keep in touch with a relative that they previously had not previously been in touch with [1]. More recent studies have contemplated the “digital divide." This divide refers to the differential between those who access the Internet and those who do not [2]. The divide has been documented in the United States as well other European countries [3,4]. Several of the Pew Research Center’s reports suggest that the digital divide is narrowing over time as the number of mobile devices increases and the broadband population becomes more diverse, especially within the African American community [5]. Lorence, however, has suggested that the divide persists with the emergence of a “digitally underserved” group [6,7].

Likewise, the impact of the World Wide Web on health care has also been radical. Consumers have flocked to the Internet to search for information on diagnoses, treatment regimes, and prognoses. Findings from the Pew Research Center’s 2008 nationally representative telephone survey suggest that 61% of Americans use the Internet to find health information, and 60% say that information they found online has impacted a health care decision they made [8]. These results have been corroborated by peer-reviewed studies. McInnes et al found that 29% of veterans had searched for health information online [9]. Health information searching was directly associated with higher levels of education, living in an urban area, and decreased health status. Lea and colleagues studied patients with head and neck cancer who received care at a comprehensive cancer center [10]. Using the computer was associated with increased educational attainment and income but younger age. Walsh conducted a survey with 1784 cancer patients and also found that use of the computer increased with education and income but decreased with age [11].

In pediatrics, there is limited information on Internet use or eHealth literacy, which is defined as the ability to “locate, evaluate, integrate, and apply information gleaned from electronic platforms” [12]. Mackert et al used quantitative methods to study eHealth literacy. They conducted focus groups with low literacy and culturally diverse parents and found that some avoided .edu or .gov websites because such websites are viewed as too complex and that some parents had expressed a lack of trust in government websites [13]. Quantitative methods have also been used. Kind et al surveyed 260 African American parents and found that greater Internet use and access were associated with higher educational attainment and income [14]. Other studies have documented parents’ Internet information seeking activities related to specific diagnoses including genetic counseling, hearing loss, and late effects of cancer treatment [15-17]. Recently, a few studies have emerged which used the eHealth Literacy Scale (eHEALS) to assess eHealth literacy in pediatrics [12]. Knapp and colleagues used this scale to measure eHealth literacy of parents whose children were enrolled in a pediatric palliative care program in Florida. Survey data from 129 parents showed that not having graduated from high school was associated with lower eHealth literacy and using the Internet as the primary information source (as opposed to their child’s physician) was associated with higher eHealth literacy [18]. Brown and Dickson used the eHEALS to assess eHealth literacy of health care students in a master’s program. They found that the students had high eHealth literacy but lacked the confidence in using information found online to make health care decisions [19]. Finally, the eHEALS was used in a 2010 intervention study to determine the intervention’s effectiveness on HIV-positive participants’ ability to access information online. The intervention was found to be associated with positive and sustaining increases in eHealth literacy [20].

Although not the focus of these few studies, it is especially important to investigate the online information seeking behaviors and eHealth literacy for the approximately 13.9% of parents in the United States whose children have special health care needs [21]. Children with special health care needs are defined as “those who have or are at increased risk for a chronic physical, developmental, behavioral, or emotional condition and who also require health and related services of a type or amount beyond that required by children generally” [21]. These children face a number of medical decisions throughout their lives, forcing their parents to routinely seek out and compile large amounts of medical information on their behalf. Oftentimes parents use the Internet to locate, process, and use information. A recent statement by the American Association of Pediatrics (AAP) urged policymakers to take into account the needs of all stakeholders when implementing online and electronic resources, including families [22]. AAP suggested that information technology education and training should be available for patients and families so that they can be involved in decision making and become empowered.

To our knowledge, no studies have sought to describe Internet use and eHealth literacy of parents of children with special health care needs. Our study addresses these gaps in knowledge. We aimed to (1) describe Internet access and use patterns among low-income parents whose children have special health care needs, (2) describe the factors associated with Internet use, (3) describe the eHealth literacy of the parental Internet users, and (4) determine which factors are associated with greater eHealth literacy. We hypothesized that there would be education and age disparities associated with Internet use and eHealth literacy.

## Methods

### Sample

Study participants were parents of children with special health care needs aged 1 to 21 years enrolled in Florida’s Children’s Medical Services Network (CMSN) program, the state’s Title V program. Title V of the Social Security Act allows for states to receive block grants to improve maternal and child health. CMSN has a network of primary and specialty care providers and each child enrolled in CMSN is assigned a care coordinator. All children with special health care needs enrolled in the program are eligible for Medicaid or the State Children’s Health Insurance Plan (SCHIP) and their families have incomes less than or equal to 200% of the federal poverty level. Children must be certified by a physician as having a special health care need.

A random sample of parents whose children were currently enrolled in CMSN were each sent a letter explaining that someone may call them to participate in the study. Telephone surveys were conducted in English and Spanish beginning in July 2009 and ending in October 2009 using the Windows based computer assisted telephone interviewing system, WinCATI (Sawtooth Technologies, Northbrook, IL). Overall, 2371 surveys were completed. The University of Florida’s Institutional Review Board approved this study. 

### Outcome Measures

The two aims of this study were to determine parents’ use of and access to the Internet, as well as the eHealth literacy of Internet users. We asked parents if they had ever used the Internet, and if so, what was their frequency of use (daily, weekly, or less often than weekly). Parents were asked where they accessed the Internet (work, home, or mobile device). To determine the eHealth literacy of Internet users, the eHEALS scale was used. The eHEALS scale measures the “ability to locate, evaluate, integrate, and apply information gained from electronic platforms’’[12]. There are eight items on the eHEALS scale that measure consumers’ perceived information technology skills. The response categories determine the level of agreement (agree, undecided, disagree) with the eight statements about online health information. Psychometric testing on the eHEALS has revealed high internal consistency (Cronbach alpha 0.88) [12]. 

### Factors

The final study aims were to explore what parent, child, and household factors are associated with Internet use and greater eHealth literacy. Several questions were included on the survey to explore these factors including parent’s age, parent’s race/ethnicity, parent’s gender, parental language spoken at home, parent’s marital status, parent’s educational attainment, type of household (single or two parent), child’s age, and child’s health status. Children’s health status was gauged by asking parents to rate their children’s health status as excellent, very good, good, fair, or poor.

### Analyses

Descriptive analyses were conducted to describe Internet use, how users accessed the Internet, and responses to the eight items from the eHEALS scale. Multivariate analyses were conducted to explore which child and household factors were associated with Internet use and greater eHealth literacy. A multivariate logistic regression was conducted to determine factors associated with Internet use. In this regression, the dependent variable was a binary indicator equal to one if parents responded that they used the Internet and zero otherwise. Finally, eight multivariate ordinal logistic regressions were performed using the responses to each of the eight statements in the eHEALS scale. Ordinal logistic was chosen because the response categories for each of the eight statements are ordinal (agree, undecided, disagree). STATA version 10.0 (StataCorp LP, College Station, TX) was used to perform the analyses [23]. 

## Results

### Sample Characteristics

Survey response rate was 58.2% (4072 had valid contact information and were approached, and, of those, 2371 completed the survey). More than one-half (1212/2371, 51%) of parents lived in a two-parent household; 1252 out of 2371 (53%) were married; 1827 out of 2371 (77%) primarily spoke English; 915 out of 2371 (39%) were white non-Hispanic; 721 out of 2371 (30%) had a high school diploma; and 2154 out of 2371 (91%) of the respondents were female (Table 1). Of all participating parents, 21% (491/2371) reported that their children were in fair or poor health, 914 out of 2,371 (39%), good health, and 943 out of 2371 (40%), excellent or very good health. Parental mean age was 40.5 (SD 10.2 years) and mean age of their children was 10.5 (SD 4.9 years).

**Table 1:** Summary statistics

Variable		n	% of 2371
**Parent's gender**			
	Female	2154	90.8%
	Male	217	9.2%
	Missing	0	0.0%
**Parent's race/ethnicity**			
	White non-Hispanic	915	38.6%
	Hispanic	688	29.0%
	African American non-Hispanic	623	26.3%
	Other	125	5.3%
	Missing	20	0.8%
**Parental language spoken at home**			
	English	1827	77.1%
	Non-English	541	22.8%
	Missing	3	0.1%
**Parent's educational attainment**			
	Less than high school	458	19.3%
	High school graduate	721	30.4%
	Some college	569	24.0%
	College graduate	597	25.2%
	Missing	26	1.1%
**Parents marital status**			
	Not married	1096	46.2%
	Married	1252	52.8%
	Missing	23	1.0%
**Type of household**			
	Single parent	1127	47.5%
	Two-parent	1212	51.1%
	Missing	32	1.3%
**Child's health**			
	Excellent/very good	943	39.8%
	Good	914	38.5%
	Fair/poor	491	20.7%
	Missing	23	1.0%

### Internet Use

Overall, 82% of all parents (1945/2371) reported that they used the Internet and 426 out of 2371 (18%) of parents reported that they never used the Internet (Table 2). Of the Internet users, about one-half accessed the Internet or email on a daily basis. Most parents had access to the Internet from home (1681/2371, 71%), 48% had access from work (1143/2371), and 43% of all parents had access from both home and at work (1015/2371). Additionally, 624 out of 2371 (26%) parents had accessed the Internet or email from mobile devices.

**Table 2:** Internet use and access

Variable		n	% of 2371
Use			
**Frequency of Internet or email use**			
	Daily	1158	48.8%
	Weekly	488	20.6%
	Less often than weekly	299	12.6%
	Never	426	18.0%
**Location**			
	Internet access from home	1681	70.9%
	Internet access from work	1143	48.2%
	Internet access from home and work	1015	42.8%
**Ever used cell phone or BlackBerry to access Internet or email**			
	Yes	624	26.3%
	No	1747	73.7%

### Multivariate Analysis: Internet Use

A logistic regression was performed where the dependent variable was equal to one to indicate Internet use and zero otherwise (Table 3). Parents of African American race, non-English speaking parents, older parents, and parents with less than a college education were less likely to use the Internet. Parents who were married and had a child with excellent or very good health were more likely to use the Internet. 

**Table 3:** Multivariate logistic regression

		(Dependent Variable: Internet Use)			
Independent variables^a^		Coefficient Estimate	95% Upper Confidence Interval	95% Lower Confidence Interval	*P* value
**Parent's gender**					
	Male	0.81	0.53	1.26	.36
**Parent's race/ethnicity**					
	Hispanic	0.78	0.50	1.20	.25
	African American non-Hispanic	0.46	0.33	0.64	< .001
	Other	1.25	0.55	2.83	.60
**Parental language spoken at home**					
	Non-English	0.42	0.28	0.62	< .001
**Parent's educational attainment**					
	Less than high school	0.06	0.04	0.09	< .001
	High school graduate	0.16	0.10	0.25	< .001
	Some college	0.32	0.20	0.52	< .001
**Parent's marital status**					
	Married	1.44	0.98	2.13	.07
**Type of household**					
	Two-parent household	1.09	0.74	1.62	.66
**Child's health**					
	Excellent/very good	1.44	1.04	1.99	.03
	Good	1.50	1.10	2.04	.011
**Age**					
	Parent's age (years)	0.94	0.93	0.95	< .001
	Child's age (years)	1.01	0.98	1.04	.48

^a^ Referent groups: female, white non-Hispanic, English speaking, college graduate, not married, two-parent household, fair/poor health

### Responses to eHealth Literacy Scale Items

Table 4 shows the response frequencies for each eHEALS item for those 1945 parents who said they used the Internet. Parents who never used the Internet were not asked the eHEALS items.

**Table 4:** Response frequencies to eHEALS items

	Agree		Undecided		Disagree	
eHEALS item	n	% of 1945	n	% of 1945	n	% of 1945
1. I know what health resources are available on the Internet.	1179	60.6%	486	25.0%	280	14.4%
2. I know where to find helpful health resources on the Internet.	1273	65.4%	371	19.1%	301	15.5%
3. I know how to find helpful health resources on the Internet.	1358	69.8%	309	15.9%	278	14.3%
4. I know how to use the Internet to answer my questions about my child's health.	1409	72.4%	297	15.3%	239	12.3%
5. I know how to use the health information I find on the Internet to help my child.	1448	74.4%	286	14.7%	211	10.8%
6. I have the skills I need to evaluate the health resources I find on the Internet.	1364	70.1%	325	16.7%	256	13.2%
7. I can tell high quality health resources from low quality health resources on the internet.	1030	53.0%	501	25.8%	414	21.3%
8. I feel confident in using information from the Internet to make health decisions.	1024	52.6%	466	24.0%	455	23.4%

Response categories are grouped into agree (including strongly agree and agree), undecided, or disagree (including strongly disagree and disagree). Missing values were recoded as undecided.

The statement “I know how to use the health information I find on the Internet to help my child” had the highest level of agreement (1448/1945, 74%). The two statements that parents had the highest level of disagreement with were related to confidence in using information received from the Internet to make health decisions (455/1945 or 23.4% disagreed with eHEALS item 8) and ability to distinguish between high and low quality information (414/1945 or 21.3% disagreed with eHeals item 7).

Although not presented in the table, bivariate analyses were conducted to determine if there were significant differences between parents who were confident in using health information versus those who were not confident and parents who were and were not able to distinguish the quality of health information. In regard to confidence in using health information, significant differences (*P* < .05) were realized. More confident parents were English speaking parents, younger parents, parents of younger children, and parents whose children had excellent to very good health versus the respective referent groups. In regard to distinguishing between high and low quality health information, significant differences (*P* < .05) were realized. Parents better able to make the distinction had higher levels of education, were younger, were parents of younger children, and had children with excellent to very good health versus the respective referent groups.

### Multivariate Analysis: eHealth Literacy

Performed were eight ordinal logistic regressions where the dependent variable represented the levels of agreement with each eHEALS statement (Tables 5). Observations where the dependent variable was missing were dropped. It is important to use this model since the response categories of agree, undecided, and disagree have an ordered nature. Results from all the eight regressions indicate that parental language, parental lower educational attainment, and older parental age were all consistently and significantly associated with lower levels of agreement with the eight eHEALS statements. For example, results from the statement “I know what resources are available on the Internet” imply that non-English speaking parents were about 35% less likely to be in a higher agreement category versus their English speaking peers. Results from the last two regressions are especially important to note given the low percentage of parents who agreed with these statement. For the statement “I can tell high quality health resources from low quality health resources on the Internet,” parents of another race, parents with less than a college degree, parents living in a two-parent household, and older parents were all significantly less likely to be in higher agreement. It is interesting that this is the only statement where non-English speaking was not significantly associated with higher agreement. For the statement “I feel confident in using information from the Internet to make health decisions,” Hispanic parents, non-English speaking parents, parents with less than a high school degree, parents who were married, parents living in a two-parent household, parents having a child with excellent/good health, and parents who were older were all significantly less likely to be in higher agreement. 

**Table 5:** Multivariate ordered logit regressions (eHEALs items 1 through 4)

		Dependent Variables							
		eHEALS Item 1		eHEALS Item 2		eHEALS Item 3		eHEALS Item 4	
Independent variables^a^		*Coefficient Estimate*	*P* Value	*Coefficient Estimate*	*P* Value	*Coefficient Estimate*	*P* Value	*Coefficient Estimate*	*P* Value
**Parent's gender**									
	Male	1.123	.49	1.107	.56	1.069	.72	0.912	.62
**Parent's race/ethnicity**									
	Hispanic	0.922	.59	1.008	.96	0.882	.45	1.063	.72
	African American	0.689	.003	0.627	<.001	0.663	.003	0.863	.30
	Other	0.659	.05	0.819	.39	0.873	.58	1.114	.67
**Parental language spoken at home**									
	Non-English	0.652	.005	0.571	<.001	0.530	<.001	0.452	<.001
**Parent's educational attainment**									
	Less than high school	0.564	<.001	0.601	.001	0.576	.001	0.492	<.001
	High school graduate	0.774	.04	0.792	.07	0.711	.01	0.710	.02
	Some college	0.869	.28	0.924	.56	0.867	.32	0.742	.04
**Parent's marital status**									
	Married	0.777	.12	0.993	.97	1.036	.84	0.838	.33
**Household type**									
	Two-parent household	0.702	.03	0.848	.32	0.862	.39	0.847	.36
**Child's health**									
	Excellent/very good	1.229	.11	1.187	.21	1.143	.35	1.185	.24
	Good	1.217	.13	1.022	.87	1.051	.72	1.061	.68
**Age**									
	Parent's age (years)	0.986	.02	0.981	.001	0.986	.03	0.978	<.001
	Child's age (years)	0.989	.34	0.988	.31	0.976	.049	0.984	.21

^a^ Referent groups: female, white non-Hispanic, English speaking, college graduate, not married, two-parent household, fair/poor health

**Table 6:** Multivariate ordered logit regressions (eHEALS items 5 through 8)

		Dependent Variables							
		eHEALS Item 5		eHEALS Item 6		eHEALS Item 7		eHEALS Item 8	
Independent variables^a^		Coefficient Estimate	*P* Value	Coefficient Estimate	*P* Value	Coefficient Estimate	*P* Value	Coefficient Estimate	*P* Value
**Parent's gender**									
	Male	1.009	.96	0.761	.12	1.047	.77	1.258	.16
**Parent's race/ethnicity**									
	Hispanic	1.089	.63	1.067	.70	1.083	.57	1.342	.04
	African American	0.949	.72	0.903	.47	0.991	.94	0.980	.87
	Other	1.073	.77	0.863	.54	0.616	.02	0.816	.33
**Parental language spoken at home**									
	Non-English	0.542	<.001	0.418	<.001	1.097	.54	0.616	.001
**Parent's educational attainment**									
	Less than high school	0.527	<.001	0.432	<.001	0.497	<.001	0.654	.003
	High school graduate	0.770	.07	0.630	.001	0.576	<.001	0.863	.20
	Some college	0.888	.43	0.758	.06	0.652	.001	0.837	.14
**Parent's marital status**									
	Married	0.761	.14	0.783	.18	0.760	.08	0.614	.002
**Household type**									
	Two-parent household	0.761	.14	0.716	.07	0.704	.02	0.656	.008
**Child's health**									
	Excellent/very good	1.326	.06	1.624	.001	1.245	.08	1.398	.008
	Good	1.078	.60	1.257	.10	1.091	.48	1.192	.16
**Age**									
	Parent's age (years)	0.979	.001	0.969	<.001	0.985	.009	0.978	<.001
	Child's age (years)	0.981	.13	0.987	.28	0.980	.06	0.992	.47

^a^ Referent groups: female, white non-Hispanic, English speaking, college graduate, not married, two parent household, fair/poor health

## Discussion

To our knowledge, our study is the first to focus on Internet use and eHealth literacy of parents of children with special health care needs. This study is unique in the population surveyed, the number of completed surveys, the ability to test for disparities, and the focus on children with special health care needs. Our study suggests that most parents have access to the Internet and use it on a daily basis, and most know how to find health information for their child. However, parents are concerned that they are unable to distinguish between high and low quality information online. Our findings expand the extant knowledge in the following ways.

First, our findings allow us to comment on access to the Internet for low-income parents of children with special health care needs. Parents of children with special health care needs are likely to have greater impetus to seek out health information compared with parents whose children do not have special health care needs. As a result, it is important that the parents’ information needs be met, and the Internet may serve as a convenient, low-cost repository of information for these parents if they have access. Our results show that 1945 of 2371 (82%) parents in our sample use the Internet and most (1681/2371, 71%) have access to the Internet at home. Compared with the Pew Research Center’s Internet and American Life Project study, which found that 57% of adults with household incomes lower than $30,000 use the Internet, parents in our study seemed to have greater Internet use [8]. Results from our study are more aligned with the Pew Research Center’s findings that approximately 74% of the general population has gone online to access the Internet, World Wide Web, or to receive email [8]. Interestingly, 624 out of 2371 (26%) parents in our sample have used mobile devices to access the Internet. Findings from the Pew Research Center’s report Mobile Access 2010 showed that 39% of the adult population are *motivated* mobile users and that African Americans and non-English speakers are among the highest users of the mobile Web [24]. Future studies should determine if the lower mobile use trends demonstrated in our sample are due to differences in income.

Second, our findings provide new insights into the factors associated with parental Internet use. Our findings corroborate Kind et al [14] who also found that higher levels of education were associated with greater Internet use for African American parents. Our additional findings are novel to the literature on parental Internet use, although they do corroborate existing information on Internet use in other subgroups. For example, we found that African American parents of children with special health care needs were less likely to have used the Internet. Although new evidence in the literature for children with special health care needs, this has been corroborated in other studies of the digital divide. Our findings concerning African American parents may be explained in part by broadband access trends. There has been a distinct gap in broadband access between African Americans and whites, although this gap has slightly narrowed in the past few years (from a 19 point gap in 2009 to an 11 point gap in 2010) [5]. Our results that older parents, less educated, and non-English speaking parents are less likely to use the Internet present opportunities for interventions. Salovey et al [25] described the creation of a community-based computer center designed to improve computer literacy of Latino and African American parents whose children were enrolled in the Head Start program. The study suggests that parental knowledge was improved. Perhaps an intervention similar to this could be used with the population in our study, parents whose children with special health care needs enrolled in Florida’s Medicaid and SCHIP programs, if these parents are interested in using the Internet, but do not have the skills to do so. Increasing Internet use for this vulnerable group is critical as many national initiatives push for the implementation of email communication between parents and providers, the adoption of electronic personal health records, and online education tools [26,27].

Third, our findings contribute to the literature on eHealth literacy. To our knowledge, our study is the first to assess the eHealth literacy of parents of children with special health care needs, and we used eHEALS, a validated survey instrument. Other studies that have explored eHealth literacy have relied on qualitative methods or nonvalidated questionnaires to understand eHealth literacy; therefore, it is difficult to compare our findings with those of other studies. However, our findings that parents may not being able to decipher the quality of online information and lack of confidence in using information to make decisions can lead to positive and negative effects on the delivery of care. For example, lack of confidence and inability to distinguish quality information may prompt parents to follow up with nurses and physicians during the health care encounter, leading to new dialogue and improved shared decision making between parents and providers. However, this may also lead to increases in competing demands on providers’ time since it would not be possible to discuss the breadth of information that parents have discovered during a single encounter. Results from the Pew Research Center indicate that 53% of adults said their most recent health search led them to seek out a second opinion or to ask their physician new questions [8]. Of course, providers routinely give educational materials to families that include links to recommended websites. Yet, for low-income families, additional interventions may be necessary such as question and answer sessions with a case manager or care coordinator to build eHealth skills and confidence. 

Fourth, results from the multivariate analysis show that there is a significant, negative association between most of the eight components of eHealth literacy and not speaking English, lower educational attainment, and being an older parent. Although these findings may not seem particularly surprising, the results of the other factors were surprising. For example, Hispanic parents are about 34% more likely than their white non-Hispanic counterparts to report higher agreement with feeling confident in using information found online. Prior evidence has noted no difference between Hispanic white and non-Hispanic white parents’ trust in physicians, and our results imply that perhaps this trust translates to online health sources whereby Hispanic parents are just as confident in what they find [28]. African American parents in our sample were significantly less likely to locate information on the Internet, but they reported no less agreement in their ability to use or distinguish the quality of information compared with white parents of children with special health care needs. Perhaps African American parents are proficient at seeking out additional information when they do not understand what they find on the Internet, or perhaps there is incongruence in what they believe they understand and what they actually do. Finally, our results suggest that parents whose children with special health care needs had excellent to good health had significantly higher agreement in feeling confident about using the information they found online versus parents whose children with special health care needs were in poor health (the referent group). Future research should focus on how interventions can be targeted to improve the confidence of this particularly vulnerable group of parents whose children with special health care needs are in poor health. Given that caregiver burden is directly associated with severity of a child’s illness, this burden may be preventing these parents from having the time to develop eHealth literacy [29]. Ironically, parents of more severely ill children may require more information due to the complexities of their children’s illnesses, and lower eHealth literacy skills may inhibit their ability to make decisions.

Several study limitations merit attention. First, although we had more than 2300 completed surveys, the response rate for the survey was 58%. While this response rate is consistent with prior surveys conducted with this population, it is possible that inherent differences between responders and nonresponders exist [30,31]. Second, our sample consists of parents whose children are enrolled in publicly funded health insurance programs. By definition, all children enrolled in the program are members of families with low incomes. Lower socioeconomic status is associated with lower health literacy, but we could not identify any studies that have investigated the effect of socioeconomic status on eHealth literacy. Both health literacy and eHealth literacy are important for utilizing Web-based applications. Our findings, which may be less generalizable to broader socioeconomic groups, show that even in low socioeconomic groups, Internet use is high (1945/2371, 82%) and that several questions on the eHEALS had high levels of agreement. Research should be conducted to determine the relationship between health literacy and eHealth literacy. Third, our study focused on parents of children with special health care needs. However, we acknowledge that parents of children without special health care needs may have different eHealth literacy and Internet use patterns. Fourth, eHealth literacy in this study was self-reported. Future research should develop a method to compare self-assessment with expert assessment in order to better interpret the results. Fifth, our study does not consider information presented to parents in multiple formats and multiple languages. Finally, we did not assess Internet use and eHealth literacy of the children and adolescents, who should also be engaged in the process of seeking health information.

Despite these limitations, our findings contribute to the dearth of evidence in the pediatric literature on parents’ Internet use and eHealth literacy. The ability to measure the eHealth literacy of parents whose children have special health care needs in an easy, valid manner as well as comment on the factors that are associated with greater eHealth literacy highlight some of the opportunities and challenges that the pediatric community faces if it wants to design Web-based applications to improve the health outcomes of children and their families. Organizations that provide information to parents on the Internet should show parents different websites to help them distinguish between high and low quality information, provide parents with information on what to look for on websites (such as citations from scientific studies), and should provide parents with information about online education and training opportunities in their area as they are available. Given the national push for health information technology adoption, understanding these issues in pediatric care is important. Future research should focus on systematically developing and testing interventions that could raise eHealth literacy and ultimately increase family empowerment. 
